# Assessing the Performance of Dried-Blood-Spot DNA Extraction Methods in Next Generation Sequencing

**DOI:** 10.3390/ijns6020036

**Published:** 2020-04-30

**Authors:** Miyono M. Hendrix, Carla D. Cuthbert, Suzanne K. Cordovado

**Affiliations:** Centers for Disease Control and Prevention; 4770 Buford Hwy, NE, Atlanta, GA 30341, USA; MHendrix@cdc.gov (M.M.H.); CCuthbert@cdc.gov (C.D.C.)

**Keywords:** next generation sequencing, DNA extraction, newborn screening, dried blood spot, cystic fibrosis, *CFTR*

## Abstract

An increasing number of newborn screening laboratories in the United States and abroad are moving towards incorporating next-generation sequencing technology, or NGS, into routine screening, particularly for cystic fibrosis. As more programs utilize this technology for both cystic fibrosis and beyond, it is critical to identify appropriate DNA extraction methods that can be used with dried blood spots that will result in consistent, high-quality sequencing results. To provide comprehensive quality assurance and technical assistance to newborn screening laboratories wishing to incorporate NGS assays, CDC’s Newborn Screening and Molecular Biology Branch designed a study to evaluate the performance of nine commercial or laboratory-developed DNA extraction methods that range from a highly purified column extraction to a crude detergent-based no-wash boil prep. The DNA from these nine methods was used in two NGS library preparations that interrogate the *CFTR* gene. All DNA extraction methods including the cruder preps performed reasonably well with both library preps. One lower-concentration, older sample was excluded from one of the assay evaluations due to poor performance across all DNA extraction methods. When 84 samples, versus eight, were run on a flow cell, the DNA quality and quantity were more significant variables.

## 1. Introduction

Molecular testing has been used with increasing frequency in newborn screening (NBS) for both primary screening (i.e., severe combined immunodeficiency (SCID) and recently spinal muscular atrophy) and second-tier testing to add clarity or specificity to the primary biochemical test (i.e., hemoglobinopathies, Pompe, mucopolysaccharidosis I, adrenoleukodystrophy and cystic fibrosis) [1,2]. The first widely adopted second-tier molecular test in the U.S. and internationally was to detect pathogenic variants that cause cystic fibrosis (CF). CF is one of the most common autosomal recessive disorders, affecting approximately 1 in 6899 births in an ethnically diverse population as measured by the California Department of Public Health Genetic Disease Screening Program Newborn Screening [3]. After the *CFTR* gene was identified in 1989 [4,5], NBS programs began adding a second-tier molecular test to detect a panel of the more common *CFTR* pathogenic variants on babies with elevated IRT to enhance the specificity of the screening results [6,7]. Since there are >2000 variants that have been identified in the *CFTR* gene [8,9], a panel of pathogenic variants will not be inclusive of all variants that can result in CF. Thus, most programs identify samples as screen positive when at least one pathogenic variant is present, resulting in a high false-positive rate due to many of the samples being carriers rather than affected by CF [10,11]. The California Department of Public Health’s Genetic Disease Screening Program was the first to redefine a screen positive as containing two pathogenic variants, by adding a third-tier screen where all babies with only one pathogenic variant had their *CFTR* gene sequenced [3]. More recently, New York State Department of Health, Newborn Screening Program adopted a similar algorithm, except, instead of using traditional Sanger sequencing, they employed next-generation sequencing (NGS) technology [12]. While CF is gateway disorder for NGS technology in newborn screening laboratories, it is anticipated that it will further be incorporated into newborn screening, particularly as more information becomes available from the National Institutes of Health funded Newborn Sequencing in Genomic Medicine and Public Health (NSIGHT) studies [13].

CDC’s Newborn Screening Quality Assurance Program (NSQAP), part of the Newborn Screening and Molecular Biology Branch (NSMBB), is mandated by Congress under the Newborn Screening Saves Lives Act to provide proficiency-testing (PT) and quality-assurance services for NBS laboratories [14]. For the molecular detection of CF, NSQAP provides a PT program to detect pathogenic variants in the *CFTR* gene that can result in CF [15,16]. CDC’s CF DNA DBS repository is made from anonymous donor-CF-patient and family-donor blood samples and contains a diverse number of *CFTR* pathogenic variants including the 23 recommended by the American College of Medical Genetics (ACMG), as well as 58 expanded pathogenic variants. These samples are characterized extensively by CDC to ensure robust performance in NBS laboratories. In quarter 1 of 2020, there were 77 participants including both domestic and international programs enrolled in the CF DNA PT program, eight of which identified that their programs use NGS technology either to sequence the coding regions of the *CFTR* gene or to identify a large number of pathogenic variants [17]. As this technology is incorporated into additional programs, it will be critical to understand how to best perform NGS in high-throughput laboratories responsible for screening entire populations of newborns while still meeting short turnaround requirements needed for NBS. In addition, it is important to thoroughly examine the DBS DNA extraction methods that are used in conjunction with NGS to ensure that they are reliable, inexpensive and practical for population-based screening in programs with varying sample loads. The CDC’s NSMBB Molecular Quality Improvement Program laboratory designed a study to evaluate the utility of nine DBS DNA extraction methods that range from a highly purified column extraction to a crude detergent-based no-wash boil prep and examines their utility in NGS using two assays to sequence the *CFTR* gene, MiSeqDx cystic fibrosis clinical sequencing assay (Illumina, San Diego, CA, USA) and Accel-Amplicon™ *CFTR* panel for Illumina platforms (Swift Biosciences, Ann Arbor, MI, USA).

## 2. Materials and Methods 

### 2.1. Samples

Twenty samples from CDC’s CF DNA DBS Repository were used in this study. These samples were collected from patients or family members affected by CF and were collected from partner CF care centers and in collaboration with the Sequoia Foundation and the California Department of Public Health. All blood was collected in EDTA blood collection tubes from adult donors with at least one pathogenic variant in the *CFTR* gene (Becton Dickinson, Franklin Lakes, NJ, USA) and shipped to CDC, where blood was spotted onto Whatman 903 filter paper (Whatman, Piscataway, NJ, USA) to create dried blood spots (75 μL) for quality assurance. Samples were selected to include a panel representing a diverse group of CF-causing variants including: R75X(c.223C>T), R117H(c.350G>A), R347P(c.1040G>C), F508del(c.1521_1523delCTT), G85E(c.254G>A), G551D(c.1652G>A), 2183AA>G(c.2051_2052delAAinsG), 3120+1G>A(c.2988+1G>A), R553X(c.1657C>T), 3849+10kbC>T(c.3717+12191C>T), 621+1G>T(c.489+1G>T), 1248+1G>A(c.1116+1G>A), 1717-1G>A(c.1585-1G>A), 2184delA(c.2052delA), R1066H(c.3197G>A), 3905insT(c.3773dupT), 394delTT(c.262_263delTT), 711+1G>T(c.579+1G>T), A455E(c.1364C>A), G542X(c.1624G>T), R560T(c.1679G>C), 2307insA(c.2175dupA), R1162X(c.3484C>T), R334W(c.1000C>T), 406-1G>A(c.274-1G>A), I507del(c.1519_1521delATC), S549N(c.1646G>A), 1898+1G>A(c.1766+1G>A), 2789+5G>A(c.2657+5G>A), 3659delC(c.3528delC), and N1303K(c.3909C>G). Note that variants 394delTT(c.262_263delTT) and 2184delA(c.2052delA) are not included in the clinical sequencing assay evaluation due to poor performance that was seen across all extraction methods. This project was approved by the institutional review boards of all participating CF care centers and the CDC’s Office of Science at the National Center for Environmental Health determined that CDC was not involved with human subjects under 45 CFR 66.012(d).b because all specimens were de-identified to CDC.

### 2.2. DNA Extraction and Quantitation

Genomic DNA used for Sanger sequence analysis was extracted from 200–250 µL whole blood (EDTA anticoagulant) using the Qiagen QIAamp^®^ DNA blood mini spin columns and re-suspended in 100 µL of Tris-buffered EDTA (TE) (Qiagen, Valencia, CA, USA). The DNA was quantified using the NanoDrop spectrophotometer (Thermo Fisher Scientific, Waltham, MA, USA) and diluted to 5–10 ng/µL for direct use for Sanger sequencing. Genomic DNA was also extracted from one 3.2 mm DBS punch using nine DBS DNA extraction methods commonly used in the NBS community. Note that there are more than these nine methods used in newborn screening laboratories. Method one: Qiagen QIAamp^®^ DNA micro columns were used according to the manufacturer’s recommendations and eluted in 20 μL of the buffer AE for the clinical sequencing assay and 50 µL of buffer AE for the Accel-Amplicon™ *CFTR* NGS panel. Method two: Qiagen Generation DNA purification and elution solutions (referred to as Qiagen purification and elution solutions) included two 15 min washes of a DBS punch using 150 µL of DNA purification solution followed by one 15 min wash with 150 µL of DNA elution solution. All washes were performed at room temperature with slight agitation and the genomic DNA was eluted from the DBS punch using 50 µL DNA elution solution with 99 °C incubation for 15 min. Method three: Qiagen generation DNA elution solution (referred to as Qiagen Elution Solution) was similar to method two except the DBS punches were washed only once with 150 µL of DNA elution solution at room temperature with slight agitation followed by an elution with 50 µL of DNA elution solution at 99 °C for 30 min. Method four: a one-step DNA extraction laboratory-developed method described by Baker et al. with modifications to the elution volume (56 µL) and incubation temperature (96 °C) and including a centrifugation step of 5 min at 3700 rpm after the final heating step [18]. This method is referred to as DNA3 solution (no wash). Method five: a similar laboratory-developed method to method four except it includes an additional wash step using 90 µL of the described solution and a centrifugation at 3700 rpm for 5 min at the beginning of the procedure. This method is referred to as DNA3 solution (one wash). Method six: a laboratory-developed method which used a TritonX-MgCl_2_ solution (referred to as TritonX-MgCl_2_ method 1) where 90 µL (10mM Tris-HCl, 0.7% potassium chloride, 0.05% MgCl_2_, 0.05% Triton-X-100) was added to a DBS punch followed by a 10 min incubation at room temperature and 40 min incubation at 99 °C followed by centrifugation at 1600 rpm for 30 min [19]. Method seven: a laboratory-developed method which used a variation of TritonX-MgCl_2_ solution (referred to as TritonX-MgCl_2_ method 2) where 60 µL of solution (10mM Tris-HCl, 0.7% potassium chloride, 0.1% MgCl_2_, 0.09% Triton-X-100) was added and incubated at room temperature for 15 min then incubated at 99 °C for 40 min followed by centrifugation at 1600 rpm for 30 min [20]. Method eight: Quanta Biosciences’ Extracta DBS solution used according to the manufacturer’s recommendation (Quanta Biosciences, Beverly, MA, USA). Method nine: Perkin Elmer’s NeoMDx™ DNA extraction kit (Perkin Elmer, Turku, Finland), as described by Gutierrez-Mateo et al., with modifications to the elution volume (70 µL), and performed as a manual extraction [21]. 

Quantification of DNA extracted from DBS punches was performed using real-time PCR of the RNase P gene (RPPH1) using the TaqMan RNaseP control reagents (Thermo Fisher Scientific) and PerfeCTa^®^ qPCR ToughMix^®^ Low ROX (Quanta Biosciences). The standard curve used for the quantification was made from human genomic DNA (Roche Applied Science, Penzberg, Germany).

### 2.3. Sequencing of CFTR 

All samples extracted from DBS were sequenced using Illumina’s clinical sequencing assay (DX-102-1001) according to the manufacturer’s procedure, except for the DNA quantity input. The protocol recommended 250 ng total DNA (5 μL at 50 ng/μL) for the library prep, however the total quantity of DNA extracted from a 3.2 mm DBS punch across all nine extraction methods ranged from 0.5 ng/µL to 14.3 ng/µL. The average DNA input was 13.1 ng (min 2.5 ng and max 71.5 ng). One sample was excluded from this assay evaluation because of poor performance across all methods (average concentration of 0.54 ng/µL) making it unsuitable for Illumina’s clinical sequencing assay. 

All extracted DNA samples were also sequenced using Swift Biosciences’ Accel-Amplicon™ *CFTR* panel for Illumina platforms where all target regions of the *CFTR* gene were amplified in a single amplicon pool that covered all exons, UTRs and regions of interest in introns 12 and 22 known to contain pathogenic variants (1811+1.6kbA>G (c.1679+1.6kbA>G) and 3849+10kbC>T (c.3717+12191C>T)). The manufacturer’s recommended DNA input was a minimum of 10 ng and the total quantity of DNA extracted from a 3.2 mm DBS punch across all nine extraction methods ranged from 0.09 ng/µL to 11.0 ng/µL. The average DNA input into the library prep was 17.4 ng (min 0.9 ng and max 110.0 ng) and was prepared according to the manufacturer’s instructions. The libraries were diluted by 1:100,000 and quantified using the KAPA Library quantification kit (KAPA Biosystems, Wilmington, MA, USA) according to manufacturer’s recommendations and run on the QuantStudio 6 or QuantStudio 12K Flex real-time PCR system (Thermo Fisher Scientific). Libraries were diluted to 4 nM and pooled together for denaturing and subsequent loading on to the MiSeq flow cell at a 20 pM final concentration. The samples were run on the MiSeqDx instrument in research mode using the MiSeq reagent kit v2 standard (300 cycles). 

Sanger sequencing was performed on all exons, intron/exon borders and a region of interest in intron 22 known to contain the CF-causing variant, 3849+10kbC>T (c.3717+12191C>T), according to the method described in Hendrix et al. [16]. 

### 2.4. Data Analysis of CFTR NGS and Sanger Sequencing 

Data from Illumina’s clinical sequencing assay were analyzed using Illumina’s MiSeqReporter v2.2.31 software. Variant calls, call rates and positions not called for each sample were used to evaluate the performance of each DNA extraction method. The metrics data for each sample were obtained by using the freeware bioinformatics tool, CoverageBED, which is part of the BEDTools suite (version 2.19.1) [22]. The CoverageBED tool uses both the binary alignment map (BAM) files (generated during the analysis of the runs) and a browser extensible data (BED) file (obtained from the instrument and is a tab-delimited text file that defines genomic regions as coordinates) to limit the analysis to the 29 target regions specified in the BED file. The average coverage for each sample was determined by taking the average coverage at each position for 19 samples, and the coverage by target region was determined by calculating the average coverage for the 19 samples for specific target regions (i.e., exons or intron regions). Data were visually inspected using Integrative Genomics Viewer (IGV version 2.4.4) from the Broad Institute [23,24]. FASTQ sequence files from the Accel-Amplicon™ *CFTR* panel runs were processed using several freeware bioinformatic tools to create a custom analytical pipeline. The first step of the pipeline was to trim the 5′ and 3′ anchored primers using Cutadapt (version 1.18) [25], using manufacturer-supplied primer-trimming files for the assay. The sequences were then aligned to the human-genome reference sequence (hg19, build GRCh37) using the Burrows–Wheeler aligner (BWA, BWA-MEM version 0.7.5a-r405) [26,27], resulting in a sequence alignment map (SAM) file for each sample. The SAM files were then converted to a BAM format, which is used for variant calling and visual inspection, using the SamFormatConverter and AddOrReplaceReadGroups tools from the Picard suite (version 2.0.1) [28]. Variant calls were made using FreeBayes, version v1.0.2 (1.2.0-2-g29c4002) [29] and Genome Analysis Tool Kit (GATK, HaplotypeCaller), version 3.5 (3.5.0-g36282e4) [30] using default conditions. The output VCF files from the variant calling were filtered with vcfintersect from the vcflib suite (version v1.0.0-rc2) [31] using a BED file supplied by the manufacturer to limit the analysis to the sequence of the areas of interest. Each variant caller produces different frequencies since they use different models and algorithms to make the calls, hence both callers were used to determine variants for the samples to increase the confidence level [32,33]. The metrics data was collected in the same manner as with the clinical sequencing assay using CoverageBED [22]. In addition, coverage uniformity was calculated using the output from CoverageBED and additional metrics data were also collected using the CollectTargetedPcrMetrics tool from the Picard suite [28] to determine the percent of aligned bases that are on-target as well as the average number of mapped reads. The data was also visually inspected using IGV.

Data from Sanger sequencing was analyzed using SeqScape software (version 3) (Thermo Fisher Scientific) with GenBank *CFTR* genomic reference sequence NG_016465 according to the method described in Hendrix et al. [16]. All analyzed NGS data were then compared for concordance against the Sanger sequence data. 

## 3. Results

Two next-generation sequencing methods using the MiSeq platform were used to characterize 20 DBS samples from the CF DNA DBS repository at CDC. Genomic DNA was extracted from a 3.2 mm DBS punch using nine different commercial or lab-developed DNA extraction methods. Traditionally, NBS laboratories do not quantify the DNA extracts prior to use in downstream assays. Rather, when the assay is validated, it is assured that it is robust enough to work with varying concentrations with a set input volume. Since this study compares DNA extraction methods using NGS procedures, concentrations were determined with real-time quantitative PCR of the *RPPH1* gene. Spectrophotometer readings were not used because DNA extracted from DBS are known to give unreliable results [34]. Illumina’s clinical sequencing assay recommended DNA input was 250 ng total DNA (5 µL at 50 ng/µL), but this quantity was not used in this study as it is not achievable from a 3.2 mm DBS punch. The average DNA input from all nine extraction methods in this assay was 13.1 ng (min 2.5 ng and max 71.5 ng). One sample was excluded from the clinical sequencing assay because of poor performance across all methods. The second NGS method, Swift Biosciences’ Accel-Amplicon™ *CFTR* panel for Illumina platforms, recommended a minimum DNA input of 10 ng with an input volume of 10 µL. The average DNA input across all nine extraction methods for the Swift assay was 17.4 ng (min 0.9 ng and max 110.0 ng). Note that no samples were excluded from the Swift assay. 

### 3.1. MiSeqDx Cystic Fibrosis Clinical Sequencing Assay 

The MiSeqDx *CFTR* Clinical Sequencing Assay is an FDA-approved assay for sequencing the *CFTR* gene, which is involved in CF. Twenty samples were extracted with nine DNA extraction methods, but one sample had to be eliminated due to poor performance across all DNA extraction methods, leaving 171 samples sequenced, which, combined, represented 37 CF-causing variants of which eight were F508del (c.1521_1523delCTT). In addition, there were 20 unique benign variants contained in several of the 19 samples tested, for a total of 141 benign variants, all of which have been confirmed with Sanger sequencing. The quality metrics for the clinical sequencing assay runs in this study had a cluster density range between 240 ± 5 to 1147 ± 15 K/mm^2^ and an average read quality score of greater than or equal to Q30 of 85.7%. All Illumina *CFTR* IVD kits, including the clinical sequencing assay tested here, come with an automated bioinformatics pipeline, which is queued upon run completion using the MiSeq Reporter application. The automated pipeline identifies sequence variants, makes the variant calls and determines call rates for the sample being interrogated. The call rate represents the percent of total sequenced bases from a sample that meet a set quality score threshold. All 171 samples sequenced had a call rate of 100% except for two where the call rate was 99.98%. In both cases, the MiSeq Reporter software could not make a call for one position in each sample. In the first sample, the variant affected was the TGpolyT region upstream of exon 10. This sample was extracted using the DNA3 solution (no-wash) extraction method. After visually inspecting the TGpolyT region using the IVG program, a manual call was accurately assigned. In the second sample with less than a 100% call rate, the position impacted was hg19 chr7:117250687 (exon 19). This sample was extracted using TritonX-MgCl_2_ method 2. Upon visually inspecting the region using the IVG program, it was found that there was an A base present at an allele frequency of 32% when the expected call was a C base. Sanger sequence confirms that this sample only contains a C base at this position, hence the presence of an A was an inaccurate finding. 

**Figure 1:**
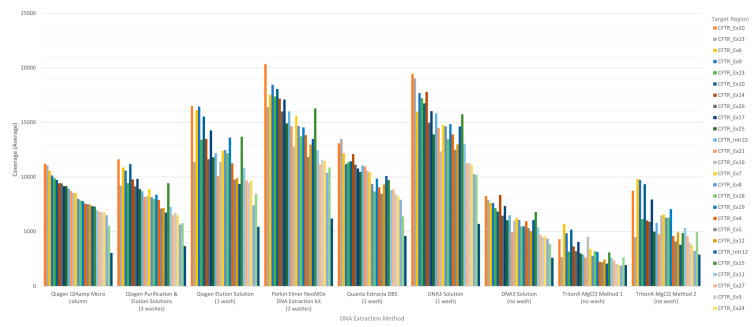
Sequence coverage (average) per *CFTR* target region for the MiSeqDx cystic fibrosis clinical sequencing assay for each DNA extraction method. Each bar represents the average sequence coverage of a single target across nineteen samples (each containing at least one pathogenic variant in the *CFTR* gene). The sequence coverage for each DNA extraction method was determined and graphed from highest to lowest using the Qiagen QIAamp^®^ micro DNA extraction method as the standard. Target regions are color coded according to the exon or intron region sequenced.

**Figure 2:**
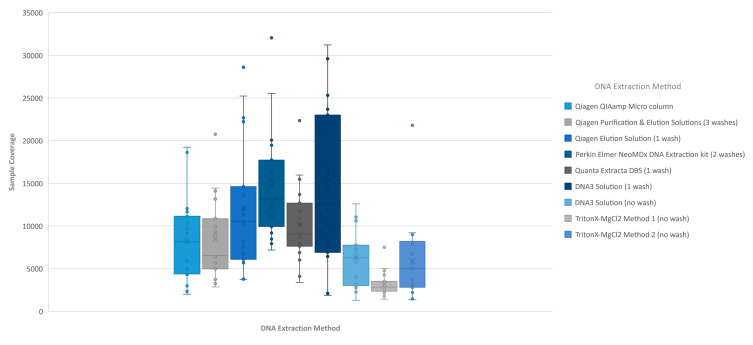
Individual sample coverage of MiSeqDx cystic fibrosis clinical sequencing assay by DNA extraction method. The coverage across all bases from an individual sample was averaged for each of the 19 samples (all of which contained at least one pathogenic variant in the *CFTR* gene). Using the box/whisker analysis, the average coverage from each of the 19 samples sequenced by the DNA extraction method was plotted to determine the upper and lower quartiles and variability between samples.

The performance of each DNA extraction in the Clinical Sequencing Assay was evaluated by assessing average coverage of the defined targets (*n* = 29 including 27 *CFTR* exons and two intronic regions) and the average coverage for *CFTR* gene sequence by base for each of the DNA extraction methods using freeware bioinformatics tools. The coverage data for both targets and *CFTR* gene sequence by base were generated by running the CoverageBED tool (part of the BEDtools suite) on the BAM file generated automatically by the MiSeqReporter software using a BED file specific to the assay in order to restrict the analysis to the regions of interest. The analysis of coverage by target region showed that the less purified and/or less concentrated DNA extraction methods including TritonX-MgCl_2_ method 1, TritonX-MgCl_2_ method 2 and the DNA3 solution (no-wash) impacted the coverage level of the 29 targets more significantly (Figure 1). All three of these methods do not include an initial wash of the DBS prior to extraction and result in a cruder prep and often lower concentration. The boil prep samples will also be sheared because the DNA was exposed to high temperatures (>95 °C) for 15 to 30 min. In addition, each of the 29 targets was in general proportionally similar between extraction methods relative to other targets in the same run (Figure 1). The analysis of coverage of the *CFTR* gene sequence by base also showed that the less purified and/or less concentrated DNA extraction methods, TritonX-MgCl_2_ method 1, TritonX-MgCl_2_ method 2 and the DNA3 solution (no wash), impacted the total coverage more significantly (Figure 2). This box/whisker analysis also allows the assessment of the variability within each extraction method across the 19 samples, since each box represents the average read coverage (depth) and the upper and lower quartile from the 19 samples by extraction method, and the whiskers indicate the variability outside the quartiles with outliers indicated by individual points. This analysis shows that the most variable of the methods within the upper and lower quartile as well as outside the quartiles was the DNA3 solution (one wash) method, whereas the least variable method was the TritonX-MgCl_2_ method 1. Since the clinical sequencing assay will result in high coverage for each sample given that the maximum sample number that can be loaded onto a single flowcell is eight, the impact of crude or low-concentration samples on the final call rate is typically negligible once the minimum specifications are achieved. A case in point is the one lower-concentration sample that was excluded from the analysis because it performed poorly across all DNA extraction methods.

### 3.2. Accel-Amplicon™ CFTR Panel for Illumina

The Accel-Amplicon™ *CFTR* panel is a commercially available NGS library preparation that is optimized to work with a variety of lower quality or quantity sample types including DBS-extracted DNA, with the optimal recommended DNA input between 10–25 ng [16]. Twenty samples were extracted with nine DNA extraction methods (*n* = 190 samples) that, combined, represented 39 CF-causing variants of which eight were F508del (c.1521_1523delCTT) and 19 unique benign variants contained in a number of the 20 samples, for a total of 123 benign variants tested, all of which have been confirmed with Sanger sequencing. Since each v2 standard flow cell was loaded with 84 samples, the results from this assay were generated on three flowcells. The quality metrics for the three runs had a cluster density of 593 ± 15 K/mm^2^, 842 ± 19 K/mm^2^ and 899 ± 24 K/mm^2^ with a read quality of greater than or equal to Q30 of 95.2%, 96.7% and 92.8% respectively. The first two runs had an average of 292,070 mapped reads with 97.3% of bases on target and 76.6% uniformity; the third run from the reformulated assay had an average of 362,411 mapped reads with 97.3% of bases on target and 75.1% uniformity. The analysis of the Accel-Amplicon™ *CFTR* panel was performed using freeware bioinformatics tools including Cutadapt (primer trimming tool) [25], BWA-MEM (sequence alignment tool) [26,27], Picard (SAMtoBAM file converter and AddOrReplaceReadGroups tools) [28] and two variant callers, FreeBayes [29] and GATK [30]. During the course of this study, the Accel-Amplicon™ *CFTR* panel was reformulated to include additional amplicons. The Quanta Extracta DBS and Perkin Elmer NeoMDx™ extraction methods were run with the reformulated assay and the Qiagen purification and elution solutions extraction was run a second time, also using the reformulated kit for comparison purposes. Using the FreeBayes variant caller, 38 of the 39 pathogenic variants and all 19 unique benign variants present across the 20 samples were called accurately. FreeBayes was unable to call the heterozygous pathogenic variant R75X (c.223C>T) contained in one of the 20 samples assayed in eight of the nine DNA extraction methods. There was one extraction method, Qiagen QIAamp^®^ micro column extraction method, where the sample containing the R75X (c.223C>T) was called correctly. When the R75X (c.223C>T) pathogenic variant was analyzed using the GATK variant caller, the one sample containing this variant was called accurately for all nine extraction methods (Table 1). The GATK results were confirmed by visually inspecting the region using the IVG program and checking for concordance with Sanger sequence data. Using the GATK variant caller, 38 of the 39 pathogenic variants were called accurately and 17 of the 19 unique benign variants present across the 20 samples were called accurately. GATK was unable to call the heterozygous pathogenic variant 2184delA (c.2052delA) contained in one of the 20 samples in three of the nine DNA extraction methods, including Qiagen QIAamp^®^ micro column, Qiagen elution solution, and DNA3 solution (no wash) (Table 1). Note this pathogenic variant was contained in the sample that was eliminated from the clinical sequencing analysis due to poor performance. When the 2184delA (c.2052delA) pathogenic variant was analyzed using the FreeBayes variant caller, the one sample containing this variant was called accurately for all nine extraction methods (Table 1). The FreeBayes results were confirmed by visually inspecting the region using the IVG program and checking for concordance with Sanger sequence data. Two benign positions, hg19 chr7:117199475 (61 bp upstream of exon 11) and hg19 chr7:117175505 (40 bp downstream of exon 6), that were not accurately called by the GATK variant caller were both called correctly by the FreeBayes variant caller as confirmed by visually inspecting the sequence using IGV and checking for concordance with the Sanger sequence data. For benign variant hg19 chr7:117199475, GATK was not able to call the variant for several samples containing this variant in three extraction methods including Quanta Extracta DBS, Perkin Elmer NeoMDx™ DNA extraction kit, and Qiagen purification and elution Solutions, and for a benign variant, hg19 chr7:117175505, GATK was not able to call the variant for one of two variant-containing samples for the Quanta Extracta DBS extraction method. 

**Figure 3:**
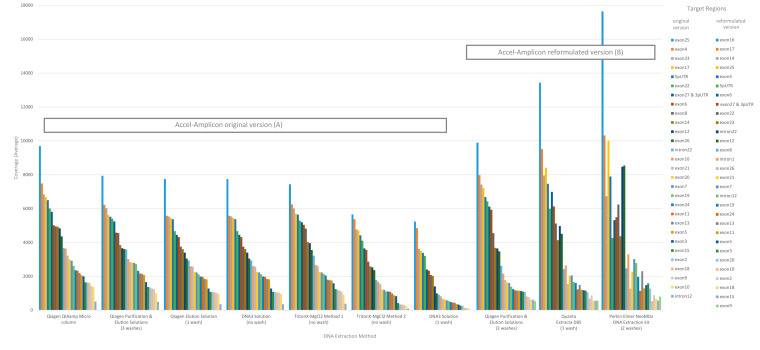
Sequence coverage (average) per *CFTR* target region for the Accel-Amplicon™ *CFTR* panel for each DNA extraction method. Each bar represents a *CFTR* target region and is the average sequence coverage across 20 samples (each containing at least one pathogenic variant in the *CFTR* gene). Target regions are color coded according to the exon or intron region sequenced. (**A**) The sequence coverage for each DNA extraction method using the original Accel-Amplicon™ *CFTR* Panel was determined and graphed from highest to lowest using the Qiagen Purification & Elution Solutions DNA extraction method as the standard. (**B**) The sequence coverage for each DNA extraction method using the reformulated Accel-Amplicon™ *CFTR* Panel was determined and graphed from highest to lowest using the Qiagen Purification & Elution Solutions DNA extraction method as the standard.

The performance of each DNA extraction in the Accel-Amplicon™ *CFTR* panel assay was evaluated similarly to the clinical sequencing assay including assessing average coverage of each target (*n* = 29 including 27 *CFTR* exons and two intronic regions in the original assay; note that the reformulated assay has an additional intronic target region and some of the original targets were extended) and the average coverage for *CFTR* gene sequence by base for each of the DNA extraction methods using freeware bioinformatics tools. The average mapped reads, uniformity and percent of bases on-target for each DNA extract were also determined using freeware bioinformatics tools. The coverage data for both targets, *CFTR* gene sequence by base, and coverage uniformity were generated by running the CoverageBED tool (part of the BEDtools suite) [22] on the BAM file generated after sequence alignment using a manufacturer supplied BED file to restrict the analysis to the regions of interest. The percent bases on-target and mapped reads were generated by running the CollectTargetedPcrMetrics tool (part of the Picard suite) [28]. In addition, the percent sequence covered at >100× was calculated since each flowcell was used to sequence 84 samples. The analysis of coverage by target region was performed on the original version of the Accel-Amplicon™ *CFTR* panel assay as well as on the reformulated version of the assay. The analysis of coverage by target region between the different extraction methods for the original version of the assay in general were similar except for the DNA3 solution (one-wash) method and the TritonX-MgCl_2_ method 2. For the reformulated version of the assay, the average target coverage was higher for all DNA extraction methods tested including the repeat of the Qiagen purification and elution solutions. Each of the 29 or 30 targets were in general proportionally similar between extraction methods relative to other targets in the same run for both the original version of the assay and the reformulated version of the assay (Figure 3).

**Figure 4:**
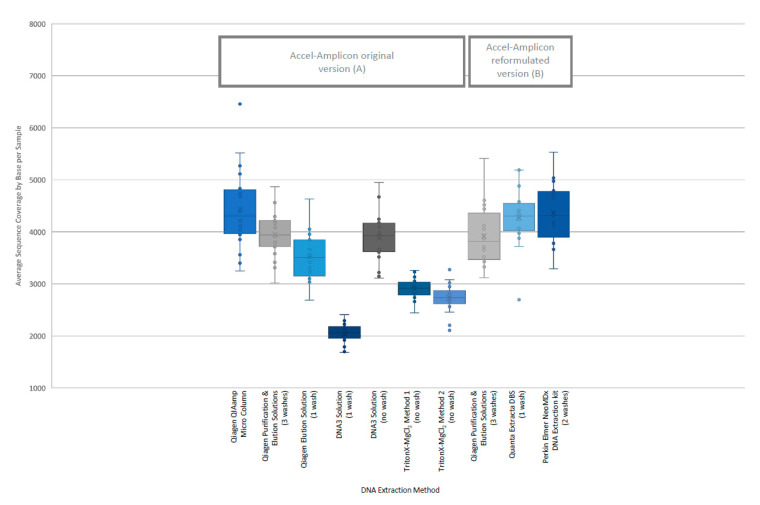
Individual sample coverage of Accel-Amplicon™ *CFTR* panel by DNA extraction method. Using the box/whisker analysis, the average coverage from each of the 20 samples (all of which contained at least one pathogenic variant in the *CFTR* gene) sequenced by DNA extraction method was plotted to determine the upper and lower quartiles and variability between samples. (**A**) The coverage across all bases from an individual sample was averaged for each of the 20 samples for the original version of the Accel-Amplicon™ *CFTR* panel. (**B**) The coverage across all bases from an individual sample was averaged for the same 20 samples sequenced using the reformulated version of the Accel-Amplicon™ *CFTR* panel.

The analysis of coverage of the *CFTR* gene sequence by base also showed that the less purified and/or less concentrated DNA extraction methods, TritonX-MgCl_2_ method 1, TritonX-MgCl_2_ method 2 and the DNA3 solution (one wash), impacted the total coverage more significantly (Figure 4). The box/whisker analysis shows that the least variable of the methods within the upper and lower quartile as well as outside the quartiles were the less purified methods including the DNA3 solution (one wash), TritonX-MgCl_2_ Method 1 and TritonX-MgCl_2_ method 2 extraction methods. While these three extraction methods were less variable, the average coverage by base was significantly less than the more purified methods. The more purified methods showed a higher coverage by base, but each had more variability between the upper and lower quartiles within the method and was consistent across the different purified methods. As with the clinical sequencing assay, more purified DNA extraction methods had an overall better average coverage. The exceptions to this generalization were the DNA3 solution (no wash) method, which behaved, more similarly to a more purified method, while the DNA3 solution (one wash) behaved more like a less purified method. 

**Figure 5:**
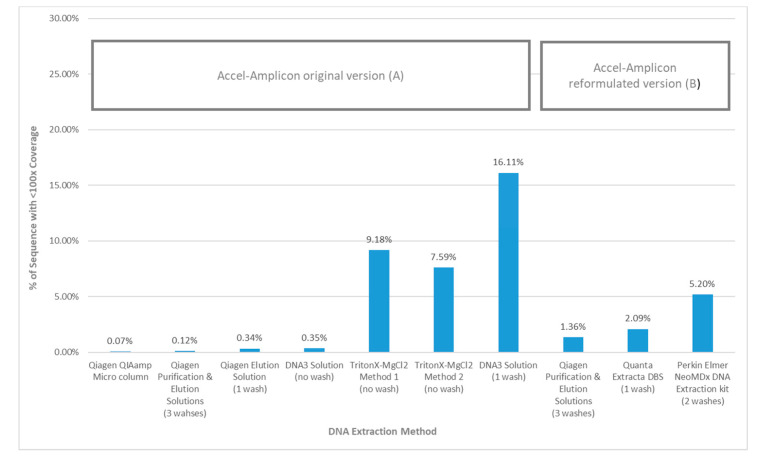
Percent of sequence using the Accel-Amplicon™ *CFTR* panel with <100× coverage by DNA extraction method. (**A**) The average coverage by base was determined for the 20 samples containing at least one pathogenic variant in the *CFTR* gene that were sequenced with the original version of the Accel-Amplicon™ *CFTR* Panel. The % of bases covered at <100× was determined for each sample and averaged across all 20 samples extracted with the same method. (**B**) The average coverage by base was determined for the 20 samples containing at least one pathogenic variant in the *CFTR* gene that were sequenced with the reformulated version of the Accel-Amplicon™ *CFTR* panel. The % of bases covered at <100× was determined for each sample and averaged across all 20 samples extracted with the same method.

When the average percent sequence covered at <100× was calculated for each DNA extraction method using the original Accel-Amplicon™ *CFTR* panel assay, the results showed that the TritonX-MgCl_2_ method 1 had the highest amount of sequence covered at <100× (16.11%) followed by the TritonX-MgCl_2_ method 2 (9.18%) and then by DNA3 solution (one wash) (7.59%). All other more purified extraction methods using the original assay had an average of 0.18% sequence covered at <100×. The DNA3 solution (no wash) method, which is less purified, was quite similar to the more purified DNA extractions when the percent sequence covered at <100× was calculated (0.35%). The average percent sequence covered at <100× was calculated for the three DNA extraction methods using the reformulated version of Accel-Amplicon™ *CFTR* panel and all three were found to be similar with an average of 2.9% of bases covered at <100×. The Qiagen purification and elution solutions method, which was the only extraction method tested with the original and reformulated versions of the assay, found relatively similar results with bases covered at <100×: 0.12% and 1.36% respectively (Figure 5).

## 4. Discussion

In this era of genomic medicine, sequencing genomes, exomes and panels of genes has become commonplace in the toolbox of the diagnostic community. NBS programs in the United States and abroad have begun embracing sequencing as a second or third-tier assay to help identify babies most at risk of diseases such as CF, Pompe, mucopolysaccharidosis type I, adrenoleukodystrophy and hemoglobinopathies. In most cases, NBS programs have utilized Sanger sequencing rather than NGS since the disorders only require laboratories to sequence a single gene, which is often not large, and the number of samples requiring sequencing are relatively few. More recently, with the adoption by a few laboratories of third-tier sequencing of the *CFTR* gene associated with CF, programs have turned to NGS since *CFTR* is such a large gene and there are greater numbers of samples requiring sequencing given the relatively high carrier rate of *CFTR* pathogenic variants. 

To support public health programs that have already adopted NGS or are considering adopting this technology, CDC did a comprehensive evaluation of DNA extraction methods using the DBS matrix and examined each extraction’s utility in two *CFTR* NGS library preparations, both of which were sequenced on the MiSeq instrument. Since the sample type in NBS is the DBS, DNA must be extracted from a punch taken from the DBS. Traditionally, most laboratories extract DNA from a 3.2 mm punch which contains approximately 3 µL of blood, resulting in a significantly lower DNA concentration than would be typical in a clinical setting. In addition, most DNA extraction methods used in public health laboratories are not highly purified; rather, they are chosen for their ease, cost and quick turnaround time due to the high-throughput requirements for NBS. In this study, DNA was extracted from 20 DBS from CF patients or carriers using nine DNA extraction methods that ranged from highly purified to crude. A set volume from each extract was input into two *CFTR* NGS library preps, based on manufacturer specifications. For the clinical sequencing assay, the average DNA input from the nine extraction methods was 13.1 ng (min 2.5 ng and max 71.5 ng), which was significantly less than the manufacturer’s recommended 250 ng. Despite the lower than optimal DNA inputs, all 20 of the CF-patient and carrier samples performed well except for one, which was excluded from the analysis due to poor performance across several extraction methods. When this sample was examined more closely, it was found that there were likely two factors, including age of the specimen and a low DNA concentration obtained in the extraction, that may have contributed to the poor performance. The sample was among the oldest in the set of 20 samples at 10.3 years old. The sample, like all others, was stored at −20 °C with desiccant. Among the 20 samples, eight were in the 10-year-old age range, and, of these, only one of the eight did not perform well, suggesting age was not the only factor that contributed to the performance issues. It is noteworthy that the seven older samples that performed well had at least twice the DNA concentration as the poor performer. While this would suggest that DNA concentration is the culprit for the poor performance, there were three of the 20 samples with a similarly low DNA concentration as the poor performer that did not suffer the same performance issues, however their ages ranged from three to eight years. Taken together, it seems like the poor performance of the one sample may have been influenced by both age and low concentration. Since it is common practice for NBS laboratories to keep samples with *CFTR* pathogenic variants long term as controls, it is important to understand the utility of these aging specimens over time and to show that most samples that are 10+ years old are still useful in this type of assay.

The clinical sequencing assay used in this study had a maximum of eight samples that could be loaded on a flow cell. Since this was a small number, the average coverage per method obtained was quite high, ranging from a high of 14,766× to a low of 3179×, depending on the DNA extraction method. In addition, many of the methods tested here were not highly purified and, in some cases, quite crude. As expected, the more crude methods that did not perform a wash of the punch prior to extraction showed the lowest average coverage, ranging between a high of 5919× for the TritonX-MgCl_2_ method 2 to a low of 3179× for the TritonX-MgCl_2_ method 1 (Figure 2). While the coverages were overall lower for the no-wash methods, the average coverage was still more than adequate to give accurate sequence data for all but one sample. Based on these results, it appears that the excess coverage was able to compensate for both the low DNA input as well as the crudeness of the DNA extractions, provided that a minimal concentration and integrity was input. Of note, this vendor will soon be marketing a new version of the clinical sequencing assay that will require a minimum of 24 samples per flow cell with a maximum of 96 samples. The capacity on the new version of the flow cell is greater, so it is not clear whether the coverage will be similar to this assay or lower than the results obtained in this study due to the increase in the number of samples. It will be important to find the maximum number of samples that can be input on a single flow cell in the new version given the less-than-optimal DNA concentration and purity from many DBS DNA extraction methods. 

Thus, the clinical sequencing assay performed remarkably well with a very high average coverage per method (>3000×) despite most DNA extraction methods being low-concentration and relatively crude. There were only two samples out of a total of 171 that had one variant each and which were not able to be automatically called by the manufacturer’s variant calling algorithm. One variant was found to be accurate after examining the sequence data with the IGV and the second was found to be inaccurate. While inaccurate data was an uncommon event, it does show that NGS can still be impacted by artifacts that are likely from DNA amplification, suggesting that confirming NGS pathogenic variants that are clinically actionable is still a good idea.

Overall, the Accel-Amplicon™ *CFTR* panel also performed very well and was able to accommodate a relatively high number of samples per flow cell (*n* = 84). This assay is noted by the manufacturer to work well with low concentration and fragmented DNA including DNA extracted from DBS by incorporating an initial amplification step. Hence, the one sample that had to be eliminated from the clinical sequencing assay was able to be included in this analysis. Because of the higher input of samples on a single flow cell, the average coverage per target was lower in comparison to the clinical sequencing assay, but still often had >1000× coverage. This assay, however, did seem more susceptible to average base coverage <100×, likely because so many more samples were input onto a single flow cell. The more-purified DNA extractions including the Qiagen QIAamp^®^ micro column extraction, Qiagen purification and elution solutions extraction, Qiagen elution solution extractions, as well as the less-purified DNA3-solution (no-wash) extraction had <0.5% of bases <100× average coverage per extraction method. Interestingly, our expectation that the DNA3 solution (one-wash) extraction method would perform better than the DNA3 solution (no-wash) was not accurate. This method had the highest rate of average bases per extraction method with <100× at 16.2%. Since the DNA3 solution has a very high pH of 11.9, it is possible that the wash, while removing some of the impurities, also impacted the DNA quality, thus possibly impacting performance. 

During this study, the Accel-Amplicon™ *CFTR* panel went through a reformulation, so there are data presented with the original version of the assay as well as the new version. The manufacturer added a new target and extended some of the target regions, which required a rebalancing of the assay. The new version of the assay showed changes to the average coverage by target and, in some cases, targets that were low or high relative to others were changed (Figure 3). Additionally, the new version of the assay also had a slightly higher <100× average coverage by base (1.36%) when looking at the Qiagen purification and elution solutions extraction method that was used in both the original and new version of the assay (Figure 5). In general, these <100× coverage regions were in intronic regions that would not impact the utility of the assay. 

The Accel-Amplicon™ *CFTR* Panel was analyzed with an in-house bioinformatics pipeline which used variant callers GATK and FreeBayes. The two callers gave different results with two pathogenic variants, R75X (c.223C>T) and 2184delA (c.2052delA), where in one case the GATK caller was more accurate and in the other case the FreeBayes caller was more accurate (Table 1). These results highlight the importance of the guidance that NGS data analysis should always be done with more than one variant caller. 

The results of this study show that many of the different DNA extraction methods for DBS that are currently used in NBS laboratories would be useful as programs begin adopting assays that involve NGS technology. As seen in our results, the extraction methods selected must be tested for specific library preps that are desired as there is unfortunately no one size fits all method. NBS laboratories are already beginning to expand their use of NGS technology beyond *CFTR* sequencing, including New York State Department of Health’s Newborn Screening Program, which has developed a targeted gene panel (*n* = 39 genes) to identify pathogenic variants causative of SCID [12], and Utah Department of Health Newborn Screening Program, which is looking at using exome sequencing with an a priori restriction to disease-causing genes that are part of their second or third-tier algorithms, in order to eliminate the need to develop different gene panels for different diseases [35]. As this expansion to integrate NGS technology into NBS uses DNA extracted from DBS, it is our expectation that for each new gene, panel of genes or even human exome, the DNA extraction and library preparation will need to be evaluated to ensure that it will give accurate and robust sequencing results. 

## Figures and Tables

**Table 1:** Accel-Amplicon CFTR assay Genome Analysis Tool Kit (GATK) and FreeBayes variant calls for R75X (c.223C>T) and 2184delA (c. 2052delA).

	R75X (c.223C>T) ^§^	2184delA (c.2052delA) ^^^		
	FreeBayes	GATK	GATK	FreeBayes
Qiagen QIAamp Micro column	29%	46%	NC ^#^	47%
Qiagen Purification and Elution Solutions	14% ^+^	46%	34%	52%
Qiagen Elution Solution	19% ^+^	49%	NC ^#^	55%
TritonX-MgCl2 Method 1	12% ^+^	48%	37%	51%
TritonX-MgCl2 Method 2	8% ^+^	33%	39%	47%
DNA3 Solution (1 wash)	9% ^+^	41%	39%	49%
DNA3 Solution (no wash)	13% ^+^	59%	NC ^#^	48%
Quanta Extracta DBS ^¥^	15% ^+^	32%	47%	51%
Perkin Elmer NeoMDx DNA Extraction kit ^¥^	9% ^+^	53%	45%	44%
Qiagen Purification and Elution Solutions ^¥^	17% ^+^	53%	50%	52%

^§^ R75X (c.233C>T) present in one sample in heterozygous state—variant caller is expected to be approximately 50%. ^^^ 2184delA (c.2052delA) present in one sample in heterozygous state—variant caller is expected to be approximately 50%. ^#^ NC-GATK variant caller threshold frequency not met to make accurate heterozygous call. ^¥^ These DNA extraction methods were tested on the reformulated version of the Accel-Amplicon CFTR Panel. ^+^ FreeBayes variant caller threshold frequency (20%) not met to make accurate heterozygous call.

## References

[1] NewSteps Resources, Newborn Screening Status for All Disorders. https://www.newsteps.org/resources/newborn-screening-status-all-disorders.

[2] Cordovado S. Sequencing in Newborn Screening Introduction and Background. https://www.aphl.org/programs/newborn_screening/Documents/2017%20Gene%20Sequencing%20Meeting/Cordovado_Intro%20and%20Background.pdf.

[3] Kharrazi M., Yang J., Bishop T., Lessing S., Young S., Graham S., Pearl M., Chow H., Ho T., Currier R. (2015). Newborn Screening for Cystic Fibrosis in California. Pediatrics.

[4] Riordan J.R., Rommens J.M., Kerem B., Alon N., Rozmahel R., Grzelczak Z., Zielenski J., Lok S., Plavsic N., Chou J.L. (1989). Identification of the cystic fibrosis gene: Cloning and characterization of complementary DNA. Science.

[5] Welsh M.J., Ramsey B.W., Accurso F.J., Cutting G.R., Scriver C.R., Beaudet A.L., Sly W.S., Valle D. (2001). Cystic Fibrosis. The Metabolic and Molecular Bases of Inherited Disease.

[6] Comeau A.M., Parad R.B., Dorkin H.L., Dovey M., Gerstle R., Haver K., Lapey A., O’Sullivan B.P., Waltz D.A., Zwerdling R.G. (2004). Population-based newborn screening for genetic disorders when multiple mutation DNA testing is incorporated: A cystic fibrosis newborn screening model demonstrating increased sensitivity but more carrier detections. Pediatrics.

[7] Gregg R.G., Wilfond B.S., Farrell P.M., Laxova A., Hassemer D., Mischler E.H. (1993). Application of DNA analysis in a population-screening program for neonatal diagnosis of cystic fibrosis (CF): Comparison of screening protocols. Am. J. Hum. Genet..

[8] Pratt V.M., Caggana M., Bridges C., Buller A.M., DiAntonio L., Highsmith W.E., Holtegaard L.M., Muralidharan K., Rohlfs E.M., Tarleton J. (2009). Development of genomic reference materials for cystic fibrosis genetic testing. J. Mol. Diagn..

[9] Tsui L., Dorfman R., Crowdy E. Cystic Fibrosis Mutation Database. http://genet.sickkids.on.ca/.

[10] Baker M.W., Groose M., Hoffman G., Rock M., Levy H., Farrell P.M. (2011). Optimal DNA tier for the IRT/DNA algorithm determined by CFTR mutation results over 14 years of newborn screening. J. Cyst Fibros.

[11] Kay D.M., Maloney B., Hamel R., Pearce M., DeMartino L., McMahon R., McGrath E., Krein L., Vogel B., Saavedra-Matiz C.A. (2016). Screening for cystic fibrosis in New York State: Considerations for algorithm improvements. Eur. J. Pediatr..

[12] Stevens C. Next Generation Sequencing in the New York State Newborn Screening Molecular Lab. https://www.aphl.org/programs/newborn_screening/Documents/2017%20Gene%20Sequencing%20Meeting/Stevens_Second%20Tier%20and%20Future%20Applications.pdf.

[13] NHGRI Newborn Sequencing in Genomic Medicine and Public Health (NSIGHT). https://www.genome.gov/Funded-Programs-Projects/Newborn-Sequencing-in-Genomic-Medicine-and-Public-Health-NSIGHT.

[14] De Jesus V.R., Mei J.V., Cordovado S.K., Cuthbert C.D. (2015). The Newborn Screening Quality Assurance Program at the Centers for Disease Control and Prevention: Thirty-five Year Experience Assuring Newborn Screening Laboratory Quality. Int. J. Neonatal Screen..

[15] Earley M.C., Laxova A., Farrell P.M., Driscoll-Dunn R., Cordovado S., Mogayzel P.J., Konstan M.W., Hannon W.H. (2011). Implementation of the first worldwide quality assurance program for cystic fibrosis multiple mutation detection in population-based screening. Clin. Chim. Acta.

[16] Hendrix M.M., Foster S.L., Cordovado S.K. (2016). Newborn Screening Quality Assurance Program for CFTR Mutation Detection and Gene Sequencing to Identify Cystic Fibrosis. J. Inborn Errors Metab. Screen..

[17] CDC NSQAP: Program Reports. http://www.cdc.gov/labstandards/nsqap_resources.html#QCReportForms.

[18] Baker M.W., Atkins A.E., Cordovado S.K., Hendrix M., Earley M.C., Farrell P.M. (2015). Improving newborn screening for cystic fibrosis using next-generation sequencing technology: A technical feasibility study. Genet. Med..

[19] Saavedra-Matiz C.A. (2016). Dried Blood Spot DNA Extraction Method Using Triton-X and MgCl_2_.

[20] Torres J. (2012). Dried Blood Spot Extraction Buffer—Includes Triton-X and No Wash. Newborn Screening.

[21] Gutierrez-Mateo C., Timonen A., Vaahtera K., Jaakkola M., Hougaard D., Bybjerg-Grauholm J., Baekvad-Hansen M., Adamsen D., Filippov G., Dallaire S. (2019). Development of a Multiplex Real-Time PCR Assay for the Newborn Screening of SCID, SMA, and XLA. Int. J. Neonatal Screen..

[22] Quinlan A.R., Hall I.M. (2010). BEDTools: A flexible suite of utilities for comparing genomic features. Bioinformatics.

[23] Robinson J.T., Thorvaldsdottir H., Winckler W., Guttman M., Lander E.S., Getz G., Mesirov J.P. (2011). Integrative genomics viewer. Nat. Biotechnol..

[24] Thorvaldsdottir H., Robinson J.T., Mesirov J.P. (2013). Integrative Genomics Viewer (IGV): High-performance genomics data visualization and exploration. Brief. Bioinform..

[25] Martin M. (2011). Cutadapt Removes Adapter Sequences from High-Throughput Sequencing Reads. EMBnet. J..

[26] Li H. (2013). Aligning Sequence Reads, Clone Sequences and Assembly Contigs with BWA-MEM. arXiv.

[27] Li H., Durbin R. (2009). Fast and accurate short read alignment with Burrows-Wheeler transform. Bioinformatics.

[28] Broad Institute, Picard Tools (Version 2.0.1), GitHub Repostiory. http://broadinstitute.github.io/picard/.

[29] Garrison E., Marth G. (2012). Haplotype-Based Variant Detection from Short-Read Sequencing. arXiv.

[30] McKenna A., Hanna M., Banks E., Sivachenko A., Cibulskis K., Kernytsky A., Garimella K., Altshuler D., Gabriel S., Daly M. (2010). The Genome Analysis Toolkit: A MapReduce framework for analyzing next-generation DNA sequencing data. Genome Res..

[31] Garrison E. Vcflib, A Simple C++ Library for Parsing and Manipulating VCF Files. https://github.com/vcflib/vcflib.

[32] Liu X., Han S., Wang Z., Gelernter J., Yang B.Z. (2013). Variant Callers for Next-Generation Sequencing data: A Comparison Study. PLoS ONE.

[33] Lefterova M.I., Shen P., Odegaard J.I., Fung E., Chiang T., Peng G., Davis R.W., Wang W., Kharrazi M., Schrijver I. (2016). Next-Generation Molecular Testing of Newborn Dried Blood Spots for Cystic Fibrosis. J. Mol. Diagn..

[34] Cordovado S. DBS DNA Extraction, Validation & Quantitation. https://www.aphl.org/programs/newborn_screening/Documents/2016%20Molecular%20Workshop/3%20-%20DNA%20Extraction%20Quant.pdf.

[35] Ruiz-Schultz N. Targeted Second-Tier Confirmatory Sequencing NBS Pipeline. https://www.newsteps.org/sites/default/files/resources/download/newsteps_new_disorders_webinar_utah_august_2018_slides_kh.pdf.

